# Using Ecological Momentary Assessment (EMA) to Assess Situation-Level Predictors of Alcohol Use and Alcohol-Related Consequences

**DOI:** 10.35946/arcr.v36.1.03

**Published:** 2014

**Authors:** Tyler B. Wray, Jennifer E. Merrill, Peter M. Monti

**Affiliations:** Tyler B. Wray, Ph.D., is a postdoctoral fellow; Jennifer E. Merrill, Ph.D., is an assistant professor; and Peter M. Monti, Ph.D., is Donald G. Millar Distinguished Professor and director, all at the Center for Alcohol and Addiction Studies, Brown University, Providence, Rhode Island.

**Keywords:** Alcohol use, abuse, and dependence, alcohol effects and consequences, alcohol-related problems, alcohol use patterns, risk factors, predictive factors, research, digital technology, electronic health technology, data collection and analysis, ecological momentary assessment (EMA), mobile electronic diary, personal data, smartphone

## Abstract

Ecological momentary assessment (EMA) has afforded several important advances in the field of alcohol research, including testing prominent models of alcohol abuse etiology in “high resolution.” Using high-tech methods for signaling and/or assessment, such as mobile electronic diaries, personal data assistants, and smartphones, EMA approaches potentially can improve understanding of precipitants of drinking, drinking patterns, and consequences. For example, EMA has been used to study complex drinking patterns and dynamic predictors of drinking in near–real time. Compared with other methods, EMA can better sample and capture changes in these phenomena that occur in relatively short time frames. EMA also has several potential applications in studying the consequences of alcohol use, including physical, interpersonal, behavioral, and legal problems. However, even with all these potential capabilities, EMA research in the alcohol field still is associated with some limitations, including the potential for measurement reactivity and problems with acceptability and compliance. Despite these limitations, electronically based EMA methods are versatile and are capable of capturing data relevant to a variety of momentary influences on both alcohol use and consequences. Therefore, it will be exciting to fully realize the potential of future applications of EMA technologies, particularly if the associated costs can be reduced.

In recent years, the rapid growth of technology has been accompanied by advances in research methodology that can lend unique insights into many critical questions about how processes involved in alcohol use and misuse unfold over time. One such technology, ecological momentary assessment (EMA), has the potential for testing and refining prominent models of alcohol abuse etiology, and in turn, for opening up new avenues for treatment.

After defining more clearly what EMA is, this article presents an overview of EMA approaches that use high-tech methods for signaling and assessment and describes how they can enhance understanding of the factors contributing to drinking, drinking patterns, and drinking consequences. It presents examples of how EMA has been used to study complex drinking patterns and dynamic predictors of drinking in near–real time, drawing on the technology’s ability to sample and capture changes in these phenomena that occur in relatively short time frames. The article further discusses applications of EMA in evaluating the consequences of alcohol use, while also highlighting key limitations of EMA in alcohol research. Finally, the article introduces exciting future applications of EMA technologies that expand the technology’s potential and reduce its costs. Although a detailed account of the many insights gleaned from high-tech longitudinal and EMA studies on alcohol is beyond the scope of this review, the aim is to discuss how technology can be used to enhance such studies and to provide examples of the dynamic phenomena relevant to alcohol use that they are able to capture. The review also focuses on drinking and consequences as they occur in the natural environment and therefore emphasizes studies of non–treatment-seeking (but at-risk) individuals, such as heavy-drinking adults, college students, and adolescents.

## What Is EMA?

The term EMA refers to a diverse family of assessment approaches that measure behavior in as close to real time as possible as participants go about their daily lives (Shiffman et al. 2008). EMA methods frequently are contrasted with global, retrospective assessment methods, which involve asking participants to recall experiences over longer recall periods, such as weeks or months. Shiffman and colleagues (2008) have described three key factors that are common to most EMA approaches: (1) Data are collected from individuals in their natural environments (representing the “ecological” component); (2) assessments are collected repeatedly over some period of time, so that fluctuation in experiences and behaviors across time and situations can be explored; and (3) assessments measure current or recent states (representing the “momentary” component). However, although these factors apply to EMA, they are not unique to this type research. Indeed, many EMA studies borrow assessment strategies from a well-established tradition of longitudinal methods, such as experience sampling, diary methods, and event-contingent responding (Csikszentmihalyi and Larson 1987; Nelson 1977), and use many of these methods in concert to assess phenomena of interest. Experience-sampling approaches, which have been in use for the last several decades, involve asking participants to report on their current experiences at certain intervals within a particular period of time. This approach aims to collect a pseudo-representative sample of experiences. Because many experience-sampling studies have incorporated devices to signal participants when it is time to complete records of their experience, these methods often are referred to as signal-contingent responding. This approach is somewhat distinct from diary, or time-contingent responding, which involves asking participants to report whether particular events, experiences, and behaviors have occurred within a certain time frame (e.g., within the past day or hours). Finally, event-contingent reporting involves collecting assessments as a particular event occurs (e.g., starting a drink).

The uniqueness of EMA methods, compared with these other approaches, may be largely historical. Some have suggested that experience-sampling approaches typically have used signal-contingent assessments to capture aspects of participants’ individual experiences, whereas EMA methods traditionally have used event-contingent assessments to primarily assess behavior (aan het Rot et al. 2012). EMA research also has been notable for its incorporation of physiological measures, such as heart rate and blood pressure (Kamarck et al. 2002). However, the distinctions between EMA and these other intensive longitudinal methods seem to be diminishing. Many modern analyses explicitly referred to as EMA studies have deployed signal-contingent assessment components to capture dynamic, rapidly fluctuating phenomena (Shiffman et al. 2008) alongside time- and event-contingent elements for capturing discrete events that can be assessed with minimal recall bias (Ferguson and Shiffman 2011). In this way, modern EMA research often uses a constellation of assessment strategies that also draw upon older traditions of intensive longitudinal research methodologies, such as experience sampling, self-monitoring, and diary approaches. Thus, the term “EMA” often is used to refer to research that incorporates various forms of these other methodologies which repeatedly assess current experiences, events, and behavior, with the goal of providing a unique window into dynamic processes and the moment-to-moment influences on these processes (Shiffman et al. 2008; Trull and Ebner-Priemer 2009; Wenze and Miller 2010).

### Electronic Technology in EMA Research

A wide variety of hardware and software technologies have been developed for conducting EMA and intensive longitudinal research. Decisions about which solutions are most fitting should be guided by the study’s hypotheses and the behaviors and experiences of interest. Because EMA studies often incorporate multiple assessment strategies, several integrated technologies have been developed to help simultaneously accomplish the many components of EMA assessments. One common set of technologies involves the use of Web-or personal-computer–based software to design the content and timing of assessments, then packaging additional software for installation on handheld electronic devices (e.g., personal data assistants [PDAs], PalmPilots^®^, and smartphones) that deploy the designated assessment components throughout the study period. This packaged software uses the features of the electronic device to signal participants to complete reports and deliver the appropriate assessments. These software packages also allow certain assessments to be initiated by participants (e.g., time-and event-contingent components). Available technologies have been discussed further by Shiffman (2007) and Connor (2013).

### Strengths of EMA

EMA methods offer a number of important advantages over more traditional global-recall methods as well as other longitudinal methods assessing broader time scales (for more detailed reviews, see Collins and Muraven 2007; Shiffman et al. 2008). First, they can characterize changes in dynamic processes occurring over relatively short time scales, particularly the dynamic interplay between various situations, environments, and behavior (Shiffman et al. 2008). As a result, they are well suited for studying how experiences and behaviors vary across contexts, change in concert with one another, and unfold over time and thus can elucidate dynamics that are difficult to characterize via retrospective recall (Shiffman 2009*a*). The intensive, repeated assessments have the extra advantage of establishing “average” levels of these dynamic constructs within participants. Consequently, the role of departures from these typical levels as situational triggers of behaviors such as alcohol use can be evaluated more clearly, without further confounding influences (Paty et al. 1992).

Second, EMA methods can avoid biases inherent in retrospective recall of alcohol use or momentary states (e.g., urges, craving, affect). Although several studies have found that EMA and recall methods produce comparable results with respect to reports of alcohol use and problems (Hufford et al. 2002; Simons et al. 2010; Wray et al. 2010), other work suggests that retrospective measures may underestimate drinking (Carney et al. 1998; Litt et al. 2000; Todd et al. 2005). However, it is important to note that discrepancies between assessment methods likely vary by the target of assessment and sample type, with retrospective reports exhibiting less bias for behaviors that occur with lower frequency and are relatively discrete (e.g., smoking or drinking) (Lewis-Esquerre et al. 2005) and in non–treatment-seeking samples (Litt et al. 2000).

Third, EMA approaches can be used to assess outcomes in high-risk groups in an ethically sound and ecologically valid way. Laboratory studies of the mechanisms of heavy drinking in certain high-risk groups, such as adolescents, often are limited because of legal and ethical concerns. EMA methods, however, allow researchers to study how adolescents experience alcohol in their natural environments, including how their subjective responses to drinking might affect drinking in the future (Miranda et al. 2014; Ramirez and Miranda 2014), supporting the utility of EMA methods for studying drinking among vulnerable and high-risk groups. Overall, EMA methodology offers particular promise as a set of tools useful for aptly characterizing momentary processes and cascades of behavior that result in high-level drinking and alcohol-related problems. For more information on EMA and its use in alcohol research, also see the article by Beckjord and Shiffman in this journal issue.

## EMA in Alcohol Research

EMA methods have been used extensively to study a variety of phenomena pertinent to alcohol use and its associated consequences. EMA methods lend themselves well to assessing drinking behavior itself, primarily because alcohol use can be organized into discrete events that are salient, easily definable, and commonly collected into “episodes” (Ferguson and Shiffman 2011). To assess alcohol use, investigators typically train participants to recognize a “standard alcoholic drink,” defined as 12 oz. of beer, 5 oz. of wine, or 1 oz. of liquor. The assessment then inquires how many of these drinks the participant has consumed over a given proximal period, such as since the last random assessment was completed, over the last several hours, or during the previous day (Simons et al. 2005; Tidey et al. 2008). This can be combined with items assessing the duration of the drinking period and the participant’s weight and sex, to calculate estimated blood alcohol content (eBAC) values that can serve as an indicator of intoxication (e.g., Piasecki et al. 2014; Ray et al. 2010). When used along with data on the timing of drinking events, this assessment approach can yield several indicators of alcohol use, such as frequency and quantity of drinking as well as level of intoxication, that can be used to study patterns of drinking over time as well as the influence of shifts in other momentary variables (e.g., mood, motivations, and context) on these patterns. Each of these areas is discussed in the following sections.

### Studying Drinking Over Time

By collecting repeated assessments over short time frames, EMA and other intensive longitudinal methods can provide a unique picture of how drinking changes over the course of a given day or a typical week and assess its day-to-day variability. For example, such analyses can help inform a better understanding of when hazardous drinking is most likely to occur. Thus far, EMA research suggests that drinking is highly situation dependent (Dvorak and Simons 2014) and that, in general, drinking episodes, and particularly high levels of use, tend to occur in the evenings on specific days, such as Thursdays, Fridays, and Saturdays (Dvorak and Simons 2014; Kuntsche and Labhart 2013). These findings are consistent with other research pointing to a “heavy-weekend-drinking” culture among college students and heavy-drinking adolescents. Within these nights, drinking also may be more likely to escalate over time on days when students have fewer obligations the following day (Kuntsche and Labhart 2013). Studies such as these can be used to identify specific times marked by high-level alcohol use to help determine optimal times for intervention. However, it should be noted that studying patterns of drinking over days and weeks often can be accomplished with methods less intensive than EMA.

### Situation-Level and Cumulative Predictors of Alcohol Use

Assessing alcohol consumption using EMA methods is particularly useful because occasions and levels of alcohol use are thought to be influenced by a variety of momentary processes. That is, many prominent etiological models of high-level alcohol use suggest that momentary fluctuations in craving (Marlatt 1978), affect (Cox and Klinger 1990), motivational factors (Cox and Klinger 1988), and social/contextual factors (Neighbors et al. 2012) can promote drinking at hazardous levels. Using EMA, fine-grained data on each of these factors can be explored both to test theories of alcohol-use etiology in the natural environment and to refine and extend these theories based on the nuances of the observed relationships. The following paragraphs discuss how EMA has been used to examine each of these factors.

#### Craving

Despite decades of research on the construct, the role of craving in high-level alcohol use is still a matter of considerable debate (e.g., Kassel and Shiffman 1992; Monti and MacKillop 2007; Tiffany 1990). Many models of craving suggest that it manifests in a subjective desire to drink that motivates alcohol use and could be a risk factor for high-level drinking (Sayette et al. 2000). Although craving has been studied extensively using animal and human experimental paradigms (Flannery et al. 2001; Monti et al. 2004), less is known about how craving accompanies drinking in the real world (Monti and Ray 2012). EMA research is well suited for evaluating a number of relevant hypotheses about the craving–alcohol use link, including whether the awareness of an urge to drink precedes drinking in people’s daily lives and whether this experience increases the likelihood of high-level use compared with low-craving occasions. In one such study using smartphones, the subjective urge to drink (measured using an 11-point visual analog scale) during the day was associated with higher levels of drinking later that day among adolescents (Ramirez and Miranda 2014). Another study of adult heavy drinkers found that craving reported during the first two drinks of a drinking episode predicted higher levels of drinking during that same episode (Ray et al. 2010).

EMA methods also have served as a useful platform for studying the effects of pharmacological treatments (e.g., naltrexone) on craving in the natural environment (Miranda et al. 2013; Tidey et al. 2008). These studies have added considerable ecological validity to existing laboratory-based studies supporting the use of these agents. Overall, the results of these studies suggest that subjective reports of craving indeed precede drinking episodes marked by high-level use and support theories of craving as an important construct in etiological models of alcohol use, as had already been proposed some time ago (Niaura et al. 1988). Moreover, this research supports the utility of using EMA methods to examine treatment effects on situational precipitants of alcohol use.

#### Stress/Affect

Both stress and affect play prominent roles in many etiological models of alcohol use. The stress-response dampening model (Sher 1987), for example, suggests that the experience of daily stressful events interacts with personal beliefs that alcohol reduces tension to predict drinking after stress. More broadly, self-medication hypotheses argue that substance use often occurs to cope with or alleviate negative affect (Khantzian 1985). Others have suggested that positive affect increases drinking, citing alcohol’s frequent use on celebratory occasions (Maggs 1999). At their core, each of these theories suggests that the experience of affect during the day has implications for decisions to drink as well as the level of use later in the day. Although many initial tests of these hypotheses have been pursued in experimental settings (Greeley and Oei 1999), more stringent tests of these models would involve examining whether people’s momentary experiences of stressful events or affect would influence drinking in the real world. However, recall methods of affective experience could be plagued by recall errors and biases (Clark and Teasdale 1982), making it difficult to accurately capture affective experiences. EMA methods can overcome these limitations by assessing stress/affect in close to real time, allowing for increased specificity about hypothesized temporal relationships involving rapidly fluctuating affective experiences and drinking.

EMA studies of stress and affect often use experience-sampling strategies to inquire about various domains of affect, assessing, for example, stress levels either “right now” or “in the last 30 minutes,” on Likert-type scales. Several items can be used to assess a specific domain of affect (e.g., level of sadness can be assessed by asking respondents if they feel sad, blue, or downhearted, whereas stress levels can be assessed using such terms as stressed or overwhelmed) to increase the reliability of each domain. In these ways, EMA methods can provide a reliable estimate of an individual’s experience of various forms of affect, sampled throughout each day.

The volume of the literature on EMA studies of stress, affect, and alcohol use prevents a thorough review (for further details, see Kassel 2009). However, some results support an association between anxiety and nervousness experienced during the day and level of drinking that night (Simons et al. 2010; Swendsen et al. 2000), whereas others suggest that anxiety may reduce the likelihood of drinking on a given night (Dvorak et al. 2014) or may be unrelated to drinking outcomes (Dvorak and Simons 2014). These mixed findings may reflect anxiety’s differential effects on drinking outcomes on a given day, such that drinking generally is less likely on days marked by high anxiety; however, if drinking does occur on high-anxiety days, the levels of drinking tend to be higher. EMA studies have provided little evidence of relationships between the experience of other forms of negative affect and stress during the day and drinking that night within non–treatment-seeking samples (Dvorak et al. 2014; Simons et al. 2010; Swendsen et al. 2000), suggesting some reservations about self-medication models. However, these findings are not universal (Simons et al. 2005). Instead, many studies consistently support associations between positive affect during the day and the likelihood of drinking, consumption levels, and intoxication that night (Dvorak and Simons 2014; Dvorak et al. 2014; Simons et al. 2005, 2010), supporting the role of positive mood and enhancement reasons in drinking as highlighted in motivational models (Cox and Klinger 1988).

#### Motivations for Drinking

A few EMA studies have more directly assessed drinking motivations as antecedents to alcohol use, following from past research suggesting that drinking for particular reasons, such as to regulate mood or facilitate social interactions, may be associated with distinct patterns of use (Cooper et al. 1992). Although these motivations traditionally are assessed by asking “how frequently” a person generally drinks for a specific reason, EMA methods also have recently been applied to assess situation-specific motives for drinking around the time when decisions to drink are made (Arbeau et al. 2011; Dvorak et al. 2014). This approach to measuring motives is more consistent with the notion that they commonly are the most proximal antecedents of drinking (Cooper et al. 1992) and reflects the fact that drinking motives frequently are situation specific (Dvorak et al. 2014). One of these studies used smart-phones to assess drinking motives on each evening when participants reported planning to drink. The assessment used single items to evaluate each domain identified in popular person-level measures of motives. For example, to determine the role of coping motives, the assessment tool asked the respondent to endorse the statement “I want to drink tonight to forget my worries, or because it helps me when I feel depressed” (Dvorak et al. 2014). The results supported associations between certain motives (i.e., drinking to enhance mood, cope with anxious mood, or facilitate social interactions) and levels of alcohol consumption on these occasions. Together, these results suggest that specific drinking motivations can meaningfully be assessed at the situation level using EMA methods and that such investigations can offer important insights into drinking for specific reasons and hazardous outcomes.

#### Social/Contextual Factors

Patterns of drinking also may change depending on the social and/or physical contexts in which drinking episodes occur. EMA methods have been used to assess these contexts and compare rates of consumption and/or intoxication across contexts. Typically, social and physical context are measured by asking participants to indicate their physical locations (e.g., work, home, school, or bar), who they are with (e.g., friends, acquaintances, family, co-workers, or alone), and whether those they are with are also drinking. In addition, EMA methods also are amenable to assessing more complex environmental and social phenomena, such as the degree to which alcohol cues are present in each physical environment (Ramirez and Miranda 2014; Scharf et al. 2013). This type of research among adult social drinkers has led to the following conclusions:

- The majority of drinking episodes seem to occur in bars, in people’s own homes, or at other people’s homes (Muraven et al. 2005; Simons et al. 2005).
- The drinker typically is with other people, notably spouses or partners, friends, and acquaintances (Muraven et al. 2005; Simons et al. 2005).
- Almost all drinking (92 percent) seems to occur around other people who are drinking (Simons et al. 2005).
- “Pre-drinking,” or beginning a drinking episode at a private residence (commonly of friends or acquaintances) before going to bars, may be associated with particularly high levels of drinking on a given night (Labhart et al. 2013).

Although EMA has been used to examine the contexts in which drinking is likely to occur, results to date are largely descriptive, and less focus has been attributed to how patterns of drinking change across contexts.

### Concurrent Smoking and Alcohol Use

Literature on the co-occurrence of smoking and alcohol use has highlighted the utility of EMA methods for assessing how these two behaviors go together and can change together over time. Using EMA, these studies have generated a number of critical insights into how alcohol use may pose relapse risks for those attempting to quit smoking (Holt et al. 2012; Shiffman et al. 2007), how the urges to drink and smoke change concurrently (Cooney et al. 2007), and how the subjective effects of co-use may reinforce future use of both (Piasecki et al. 2011). Such research has capitalized on the strengths of EMA methods for helping understand the moment-to-moment interplay between the precipitants and effects of another substance (e.g., tobacco) when used along with alcohol.

### Conclusions

Taken together, the above studies demonstrate that electronically based EMA methods are versatile and can capture data relevant to a variety of momentary influences on alcohol use, including shifts in craving/urges, stress, affect, drinking motivation, and environmental/contextual factors. These data present a more fine-grained picture of the situational factors that may be differentially associated with particular patterns of use (e.g., high-level intoxication) in both adult and adolescent heavy drinkers.

## Using EMA to Examine Predictors of Problems/Consequences

In addition to measuring alcohol use, a handful of studies also have used EMA methods to examine alcohol-related problems/consequences in close to real time. This perspective is important because it affords a potentially more accurate insight into how both fluctuating precipitating factors and particular patterns of use contribute to the development and maintenance of alcohol-related problems. Understanding the experience of alcohol-related problems in real time also may provide a more stringent test of various etiological models of the development of alcohol use disorders (AUDs) in the real world. Indeed, many of these models attempt to describe how alcohol-use behavior translates into the inability to control drinking, in spite of the desire to do so, or the experience of problems because of use (Marlatt et al. 1988).

To assess consequences of drinking in an EMA framework, popular person-level measures of alcohol-related problems, such as the Rutgers Alcohol Problem Index (RAPI) (White and Labouvie 1989) and the Drinker Inventory of Consequences (DrInC) (Miller et al. 1995), commonly are abbreviated and presented in checklist form. These measures ask participants to indicate whether they have experienced any of the problems presented within a specified window of time, such as within the last 30 minutes in the case of experience sampling or within the previous day in the case of self-monitoring. Using this approach, investigators can assess various domains of alcohol-related problems, including physical symptoms, interpersonal problems, behavior dysregulation, legal problems, and more (Simons et al. 2005). As expected, findings from such EMA studies of alcohol-related problems generally suggest that heavier levels of consumption on a given night are associated with experiencing more alcohol-related problems that night (Dvorak et al., 2014; Simons et al. 2005, 2010).

Other studies have examined both within-person influences on alcohol problems and between-person influences on the real-time link between event-level use and problems. With respect to within-person influences, experiencing certain types of negative affect during the day (e.g., irritability, loneliness, boredom, or nervousness) has been shown to directly increase the risk for experiencing general alcohol-related problems that night, regardless of a person’s level of drinking that night (Simons et al. 2005). Predicting acute dependence symptoms, Dvorak and colleagues (2014) also showed that the experience of anxiety during the day was related to drinking specifically to cope, which in turn was associated with higher levels of drinking and symptoms of dependence on a given night. Together with other studies, these findings suggest that while experiencing particular forms of negative affect during the day may not universally increase alcohol use later that night, it may increase the risk for experiencing consequences among those who do drink, providing some support for self-medication and coping models of AUD etiology (e.g., Cox and Klinger 1988).

Regarding between-person effects on the association between drinking and problems, one study showed that people with lighter overall drinking patterns may experience more acute dependence symptoms on a given night when intoxicated, compared with people with higher levels of overall drinking (Simons et al. 2010). Another investigation demonstrated that higher levels of impulsivity were associated with stronger associations between both drinking and problems and negative affect and problems (Simons et al. 2005). In combination with baseline assessments of individual differences, EMA offers a unique opportunity to understand how factors specific to a person (e.g., drinking pattern or personality) may influence moment-to-moment associations between alcohol use, other affective or situational states, and the sum of consequences experienced during a drinking episode.

Electronic-diary technologies also have been used to examine specific types (rather than the sum of) alcohol consequences, including hangovers and intimate partner violence (IPV) (Moore et al. 2011; Piasecki et al. 2010). These studies largely have used electronic diaries to establish temporal relationships between alcohol use and each of these outcomes. For example, Piasecki and colleagues (2010) found that endorsement of hangover-like experiences as assessed by the Hangover Symptom Scale (Slutske et al. 2003) increased as a function of number of drinks consumed the evening before; this association was weaker for males and did not vary by smoking status. In other work, IPV was assessed by asking whether various categories of IPV, derived from the Conflict Tactics Scale, occurred with the participant’s partner on the previous day (e.g., “insulted or swore, pushed or shoved”) (Moore et al. 2011). In this study, the level of alcohol use before or during these events increased the odds of IPV. Thus, these results suggest that heavy alcohol use may increase the risk for experiencing specific interpersonal problems; however, the effects of alcohol on risky sexual behavior in particular may vary according to person-level factors.

## Limitations and Measurement Issues in EMA Research

As described in this review, electronic EMA methods are versatile and flexible tools for measuring momentary phenomena of interest to alcohol research. Like any other research method, however, these methods are associated with important potential limitations despite their strengths. These include, but are not limited to, the potential for measurement reactivity, problems with acceptability and compliance, and issues with missing data.

Behavioral reactivity, or the change in a person’s behavior when being monitored (i.e., the “observer effect”), is a potentially important threat to the validity of findings from EMA and intensive longitudinal studies. However, the impact of reactivity effects likely depends on a number of factors. For example, reactivity may be more pronounced among those who are highly motivated to report changes in their drinking. Indeed, several studies have demonstrated reactivity effects in samples of heavy drinkers both in treatment and in the community at large (Collins et al. 1998; Litt et al. 1998). However, these results are contrasted with at least one study that found minimal evidence of reactivity among college students (Hufford 2002). Some have also suggested that reports of certain behaviors or experiences inherently may be affected because participants are tasked with monitoring them; for example, subjective feelings of craving potentially may increase when participants attend to craving cues (Tiffany 1990). Finally, other characteristics of study design and measurement also may affect reactivity. Thus, lengthier monitoring periods and time-intensive assessment methods (e.g., paper-and-pencil methods) may enhance the potential for reactivity. To date, scant empirical data are available on the influence of these factors on reactivity, leading to suggestions that uniform procedures should be used routinely by researchers to assess and report reactivity in studies using intensive longitudinal methods (for further details, see Barta et al. 2012).

Some investigators have expressed concerns about the burden placed on participants through EMA-based protocols, suggesting that the demands on participants may result in both poor acceptability and compliance. Again, these effects may vary across study characteristics, including the length of the assessment period, intensity of assessment approach, participant compensation schedule, and strategies for “coaching” participant compliance. Although compliance rates for some assessments have been high in many studies (e.g., 84 to 85 percent) (Piasecki et al. 2012; Simons et al. 2010), several EMA studies that do not support good adherence undoubtedly remain unpublished. Thus, although published studies show that EMA methods can be generally acceptable and tolerated, alcohol researchers should be mindful that the increased specificity offered by these methods may come at a cost and that in some cases, less intensive methodologies that achieve the study goals and cost less may be preferable.

Measurement reactivity, poor compliance, and other issues also pose significant challenges for the analysis of EMA data. In particular, problems with compliance with the numerous repeated assessments common to many EMA protocols raises concerns about the preponderance of missing data that these methods sometimes produce. Moreover, EMA studies of alcohol use involving event-contingent reports (e.g., when participants are asked to report each drink) could have similar limitations because respondents may withhold information deliberately, so that reports may not be missing at random. Both of these issues have implications for the assumptions of specific statistical models. Even when using compliance maximization procedures to reduce missing data, investigators must understand the nature of missing data when choosing an analytic approach for EMA data and ensure that it exerts minimal impact on both the analyses employed and the conclusions drawn from the study (Sokolovsky et al. 2014). Further details on analytic issues involved in EMA research have been reviewed by Shiffman (2014).

## Future Directions for EMA-Based Alcohol Research and Conclusions

Despite their current limitations, EMA methodologies offer considerable promise for studying a variety of questions about how the process of using alcohol and experiencing its consequences unfolds in the real world. In particular, several exciting advances in technology-mediated EMA approaches may further expand the potential of such studies. One of these is the use of mobile devices for delivering and collecting data on reaction-time–based behavioral tasks relevant to alcohol. This concept already has been employed in smoking studies (Shiffman et al. 1995; Waters and Li 2008). Incorporating behavioral tasks into EMA protocols designed to understand alcohol use and its consequences would allow exciting new views into momentary changes in constructs measured by reaction time (e.g., executive/inhibitory control, implicit processes) that occur as a result of “natural” phenomena.

There are also considerable opportunities for integrating physiological measures into existing EMA protocols. “Ambulatory monitoring” and other methods of real-time physiological data present exciting opportunities to expand the utility of EMA methods, particularly in reducing the number of self-reports and thus participant burden (Kumar et al. 2013). A more extensive review of biochemical monitoring is presented in the article by Greenfield and colleagues in this journal issue.

Finally, EMA methods may potentially inform the development of interventions that can be delivered at the times and in the contexts they are most needed (i.e., context-sensitive and “just-in-time” interventions). These interventions, which use the familiar modality of smartphones, represent an exciting new avenue for helping individuals reduce hazardous drinking and its related consequences (Kumar et al. 2013; Lathia et al. 2013). Such interventions are reviewed more extensively in the article by Beckjord and Shiffman in this issue.

Overall, technology-mediated EMA methods have allowed a number of important advances in the field of alcohol research. For example, they have offered researchers the opportunity to refine models of alcohol use to achieve a more ecologically valid view of this process as it unfolds in the natural world. Rapid advances in technology, when integrated with EMA methods, also are gradually enabling researchers to bring their questions out of the laboratory and into the real world. Indeed, the advancement of technology will soon enable alcohol researchers to test many key hypotheses with optimal ecological validity.

## Figures and Tables

**Figure:**
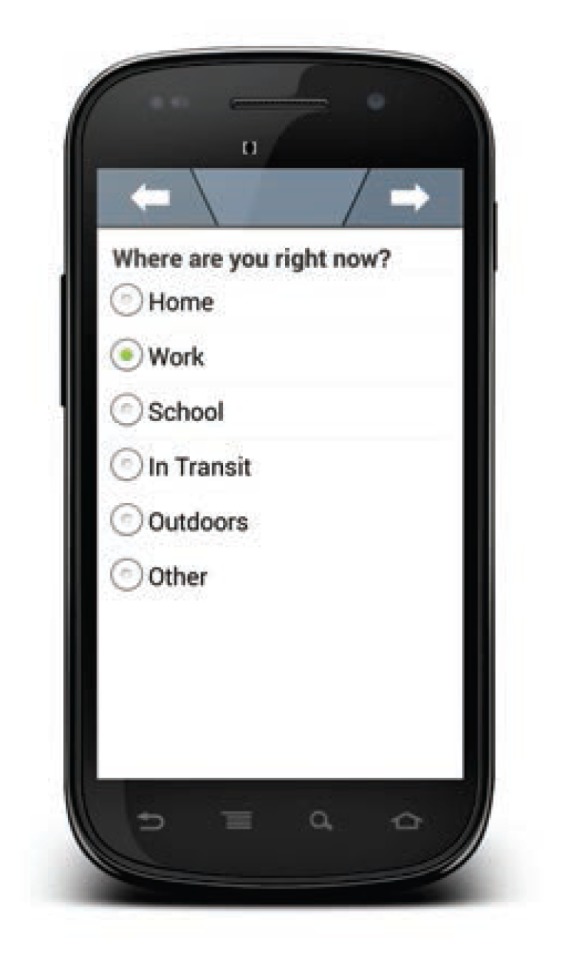
Screenshot of a typical question used during user self-report of alcohol consumption in an ecological momentary assessment (EMA) approach to monitoring alcohol consumption. NOTE: ^©^movisens GmbH
